# The Omental Cake Sign in Pediatric Tuberculosis

**DOI:** 10.3390/diagnostics12112754

**Published:** 2022-11-10

**Authors:** Giulia Fichera, Francesco Causin, Diego Cecchin, Chiara Giraudo

**Affiliations:** 1 Unit of Pediatric Radiology, University Hospital of Padova, 35128 Padova, Italy; 2 Nuclear Medicine Unit, Department of Medicine—DIMED, University of Padova, 35122 Padova, Italy; 3 Unit of Advanced Clinical and Translational Imaging, Department of Medicine—DIMED, University of Padova, 35122 Padova, Italy

**Keywords:** omental cake sign, abdominal tuberculosis, children, radiology

## Abstract

Ultrasound and computed tomography (CT) images showing ascites and omental infiltration (omental cake sign) in a 12-year-old girl with abdominal pain and fever for two weeks. The presence of abdominal and mediastinal lymphadenopathy as well as of a pulmonary consolidation at CT suggested a diagnosis of tuberculosis which was then clinically confirmed. After treatment with ethambutol, rifampicin and isoniazid, pyrazinamide, and vitamin B6 (i.e., intensive treatment for two months followed by a continuation phase with two drugs regimen for four months) the patient fully recovered. Abdominal involvement is rare in children with tuberculosis but the presence of omental involvement together with ascites and enlarged lymph nodes at imaging may suggest this diagnosis and guide the clinicians to proper testing.

A 12-year-old girl from Kosovo was referred to our tertiary pediatric center for fever (39.2 °C) and abdominal pain for two weeks. Blood tests showed low levels of hemoglobin, high levels of C-reactive protein (104 g/L and 277.40 mg/L, respectively), and high monocytes count (1.08 × 10^9^/L). Her current and past clinical history was otherwise unremarkable except for a recent close contact to an uncle with pneumonia. Given the symptoms, the child underwent abdominal ultrasound which demonstrated omental thickening and infiltration (i.e., omental cake sign) [1] as well as ascites (Figure 1a and b, respectively). Due to these findings, to better characterize whether it was an infectious or a neoplastic disease, a whole-body contrast enhanced computed tomography (CT) was performed. The CT scan showed enlarged lymph nodes in the abdomen (Figure 2a) and at the right pulmonary hilum, at this latter level with necrotic core (Figure 2b). Moreover, a pulmonary consolidation in the right upper lobe was identified (Figure 2c).

The radiological findings were highly suggestive for tuberculosis which was then confirmed with both tuberculin skin and Quantiferon blood tests (mitogen value 4.082 UI/mL; threshold > 0.50). She was then treated with ethambutol 400 mg, rifampicin and isoniazid 150 mg, pyrazinamide 500 mg (i.e., intensive treatment for two months followed by a continuation phase with two drugs regimen for four months), and vitamin B6 300 mg. Although the patient left the country shortly after, we have been notified that after therapy she fully recovered.

Abdominal tuberculosis is rare in children, especially in absence of other debilitating diseases and it occurs in the 0.1–3.5% of patients with pulmonary infection [2]. As demonstrated in the literature, ascites, lymphadenopathies, and peritoneal thickening are among the most common findings of abdominal involvement [3,4,5,6]. In particular, the omental cake sign, as in our case, is characterized by an inflammatory thickening of the omental fat and usually represents an advanced disease. In children, most of the times omental cake is caused by infections like tuberculosis or histoplasmosis, mimicking peritoneal carcinomatosis, while it is rarely associated with cancers like lymphoma, rhabdomyosarcoma, Wilms tumor or neuroblastoma [7,8,9].

From a radiological point of view, the distinction between malignant and benign omental cake might be quite challenging. Some authors, for instance, suggested that mesenteric nodules, nodular or symmetrical thickening of the peritoneum, splenic calcifications, and splenomegaly may suggest tuberculosis [7,8,9,10]. Nevertheless, some of these features were not evident in our patient, demonstrating how this diagnosis might be challenging.

Despite a reported trend of decline of the rate of tuberculosis [11], there is the need to increase awareness about this infection in the pediatric population, not only among radiologists. In this direction, the Global Tuberculosis Programme of the World Health Organization, established in 1997, recently highlighted the importance of the systematic collection of information regarding tuberculosis in children [12].

As a result of increased reporting coverage and more accurate data collection, it became clearer that children younger than 5 years are at higher risk of severe infections, and that the lack of a correct diagnosis and therefore of a proper treatment are associated with higher mortality rates (22% in non-treated children vs. 9% in treated ones) [12]. In line with this evidence, the model recently proposed by Yerramsetti et al. showed how pediatric tuberculosis is often under-reported and underlined the importance of easy diagnostic tools, especially in high-burden areas [11].

Radiologists can certainly play a role in such settings by improving the diagnostic process of tuberculosis in children, even in cases without immunodeficiencies or debilitating diseases.

Thus, pediatric radiologists identifying omental cake, ascites, and enlarged lymph nodes with or without pulmonary signs, should include tuberculosis among their differential diagnoses and guide their clinicians to proper testing.

## Figures and Tables

**Figure 1:**
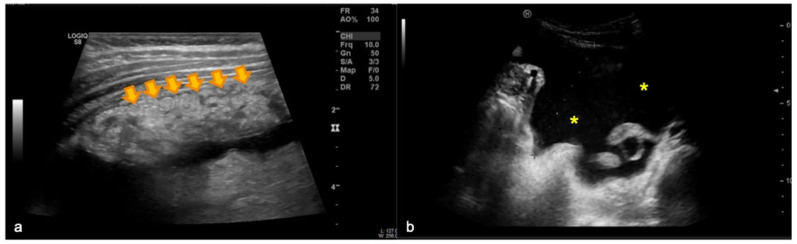
Abdominal ultrasound images of the 12-year-old patient showing the omental cake sign characterized by omental thickening and infiltration (orange arrows in **a**) and ascites (yellow asterisks in **b**).

**Figure 2:**
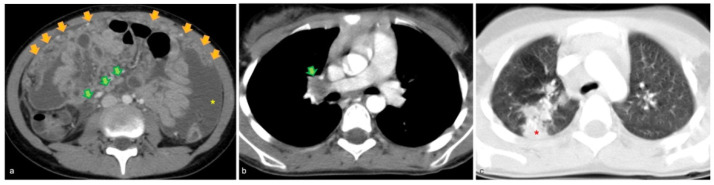
Axial images of the contrast enhanced whole-body computed tomography scan of the same child confirming the omental cake and ascites (orange arrows and yellow asterisk in **a**), enlarged abdominal and mediastinal lymph nodes (green arrows in **a**,**b**) with a necrotic core in this latter area, and a pulmonary consolidation in the right upper lobe (red asterisk in **c**).

## Data Availability

Not applicable.
